# Tackling the tumor microenvironment: what challenge does it pose to anticancer therapies?

**DOI:** 10.1007/s13238-014-0097-1

**Published:** 2014-09-05

**Authors:** Fei Chen, Xinyi Qi, Min Qian, Yue Dai, Yu Sun

**Affiliations:** 1 Institute of Health Sciences, Shanghai Institutes for Biological Sciences, Chinese Academy of Sciences, Shanghai, 200031 China; 2 School of Medicine, Shanghai Jiao Tong University, Shanghai, 200025 China; 3 VA Seattle Medical Center, Seattle, WA 98108 USA; 4 Department of Medicine, University of Washington, Seattle, WA 98195 USA

**Keywords:** tumor microenvironment, DNA damage, secretory phenotype, therapy resistance, genotoxicity, clinical intervention

## Abstract

Cancer is a highly aggressive and devastating disease, and impediments to a cure arise not just from cancer itself. Targeted therapies are difficult to achieve since the majority of cancers are more intricate than ever imagined. Mainstream methodologies including chemotherapy and radiotherapy as routine clinical regimens frequently fail, eventually leading to pathologies that are refractory and incurable. One major cause is the gradual to rapid repopulation of surviving cancer cells during intervals of multiple-dose administration. Novel stress-responsive molecular pathways are increasingly unmasked and show promise as emerging targets for advanced strategies that aim at both *de novo* and acquired resistance. We highlight recent data reporting that treatments particularly those genotoxic can induce highly conserved damage responses in non-cancerous constituents of the tumor microenvironment (TMEN). Master regulators, including but not limited to NF-kB and C/EBP-β, are implicated and their signal cascades culminate in a robust, chronic and genome-wide secretory program, forming an activated TMEN that releases a myriad of soluble factors. The damage-elicited but essentially off target and cell non-autonomous secretory phenotype of host stroma causes adverse consequences, among which is acquired resistance of cancer cells. Harnessing signals arising from the TMEN, a pathophysiological niche frequently damaged by medical interventions, has the potential to promote overall efficacy and improve clinical outcomes provided that appropriate actions are ingeniously integrated into contemporary therapies. Thereby, anticancer regimens should be well tuned to establish an innovative clinical avenue, and such advancement will allow future oncological treatments to be more specific, accurate, thorough and personalized.

## Introduction

There are obstacles in clinical cancer therapies. For years, solid tumors account for the major burden to human health, and epithelial cancers arising in tissues including lung, breast, prostate, colon, ovary and pancreas constitute approximately 80% of all cancers (Visvader and Lindeman, 2008). Solid tumor formation involves the co-evolution of neoplastic cells together with extracellular matrix and stroma that covers tumor vasculature and immune cells (Junttila and de Sauvage, 2013). Significant progress has been made in clinical treatments, particularly DNA damage-oriented chemotherapy and radiotherapy. However, vast majority of these therapeutic regimens ultimately fail to cure patients, and even tumors that show dramatic initial responses to therapy frequently relapse as resistant malignancies.

**Figure 1:**
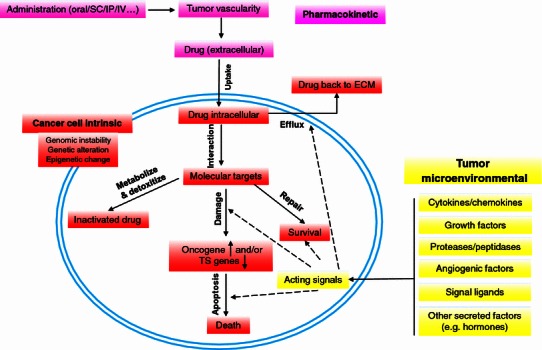
**Schematic paradigm of resistance mechanisms in cancer treatment**. Mechanisms underlying cancer therapy failure and treatment resistance are summarized. Factors implicated in either pharmacokinetic, cancer cell specific, or tumor microenvironmental categories have functional roles in treatment-induced responses. Changes in intracellular active drug concentrations, drug-target interactions, target-mediated cell damage, damage-induced cell death machineries or the signals from extracellular environments are actively at play under *in vivo* conditions. In contrast to other factors, the tumor microenvironment contains diverse stromal cell types (fibroblasts, smooth muscle cells, immune cells, endothelial cells, neuroendocrine cells, adipocytes, and pericytes) and comprises a large body of cytokines, chemokines, growth factors, proteinases, and hormones, most of which are signaling ligands, can impact pathophysiological responses to anticancer agents. Thus, the central determinants of therapeutic outcome may be highly dependent upon paracrine survival or stress signals. It is well documented that gene function and relevance varies remarkably when compared *in vivo* and *in vitro*, and studying the effect of defined genetic alterations on therapeutic response in either native or damaged tumor microenvironment is critical for effective drug development, personalized anticancer regimes, and optimal design of combination therapies. Colored text boxes: pink, pathways of drug actions; red, processes occurring in cancer cells during disease progression; yellow, signals generated by the tumor microenvironment. SC, subcutaneous injection; IP, intraperitoneal injection; IV, intravenous injection; ECM, extracellular matrix; TS, tumor suppressor

Although the standard care for cancer patients is usually a combination of surgery and DNA damaging therapy for cytoreduction or cytostasis under pathological circumstances, drug sensitivity is compromised in almost all patients with metastatic diseases. Reasons for such “apparent drug resistance” can be classified into three categories: pharmacokinetic, cancer cell innate and microenvironmental. A basic model of the functional roles of these interactive mechanisms is illustrated in Fig. 1. At molecular levels, although resistance is usually a consequence of cancer cell intrinsic genetic changes including enhanced genomic instability and mutagenesis, epigenetic alterations, decreased oxidative stress, presence of multiple drug resistance (MDR) transporters and up-regulation of drug efflux pumps (Wang and Chen, 2013; Goruppi and Dotto, 2013), an emerging body of studies pinpoints that resistance to cancer therapies is also conferred by cell extrinsic factors such as cytokines, growth factors, proteases and other soluble ligands generated from the TMEN (Campisi, 2013). These factors play key roles in regulating tumor cell proliferation, survival and malignancy through the activation of diverse signaling pathways, including the Smad, PI3K, Jak/Stat, NF-κB, MAPK, CXCR2 and IL-1 network (Nguyen et al., 2009; Ohanna et al., 2011; Coppé et al., 2011).

While many studies have addressed the activities of tumor-proximal factors in cancer progression, relatively few have clarified the role of TMEN in therapeutic outcome. We hereby review that a unique feature of damaged cells resulting from DNA-targeting therapeutics, namely cell non-autonomous secretion, may contribute major extrinsic force towards acquired resistance of cancer cells. The remarkable secretory responses of such damaged cells, mainly stromal cells residing in TMEN, may occur in numerous situations. Thus, genotoxic approaches that cause DNA damage response (DDR), unexpectedly, trigger prosurvival signaling in a neoplastic or pre-neoplastic context by providing a micro-reservoir of various pathophysiological conditions that harbor occult arsenals and subsequently fuels tumor relapse. How to overcome these obstacles to successful anticancer treatments remains an increasingly hot topic and requires intelligent inputs from both basic and translational perspectives.

## Therapy-triggered damage, cellular senescence and secretory phenotype

In clinical oncology the most prevalent non-surgery cancer treatments are radiation and chemotherapy, with a shared rationale to generate DNA damage (Jackson and Bartek, 2009; Tell and Wilson, 2010). The premise is that most cancer cells are DDR-dysfunctional, of higher rate metabolism and faster proliferation than most normal cells, with their S phase particularly vulnerable to DNA insults (Pulukuri et al., 2009). Multiple chemotherapeutic drugs are in pre-clinical and clinical trials with an aim to chemosensitize and radiosensitize cancer cells, but they also cause comprehensive senescence, as an alternative to or possibly overlapping with dominant cellular outcomes such as apoptosis, autophagy or mitotic catastrophe. Drug-inducible senescence acts as a cellular effector program and can be induced *in vitro* by a range of biochemically unrelated DNA damaging anticancer moieties, such as DNA topoisomerase I inhibitor camptothecin, the topoisomerase II inhibitor doxorubicin, the cross-linking agent cisplatin, or to a lesser extent, the anti-metabolite cytarabin. In contrast, some anti-microtubule agents that do not damage DNA such as docetaxel, vincristine or vinblastine basically cause senescence but fail to produce a secretory phenotype (unpublished). It is conceivable that cancer cells in a senescence-like state might remain as “dormant” tumor cells and therefore represent a dangerous potential for tumor relapse (Collado and Serrano 2010). More recently, the biopsy analysis of prostate cancer (PCa) patients demonstrated that a phase II clinical trial of mitoxantrone-involved neoadjuvant chemotherapy induces a typical senescence phenotype followed by the development of secretory features (Sun et al., 2012). Thus, cellular senescence is an early response upon stress and among the biological consequences of genotoxic damage to cells *in vivo*. A representative list of chemicals that can generate a DDR thereby triggering cellular senescence either *in vitro* or *in vivo* is provided in Table 1. Although a beneficial cell-autonomous role of senescence is to facilitate the clearance of senescent cells by the immune system involving inflammatory cells such as macrophages, phagocytes and natural killer (NK) cells, active products of these cells are biologically deleterious and can drive various degenerative pathologies, the most deadly of which is cancer (Chandler and Peters, 2013; Campisi, 2014; Pallasch et al., 2014).

**Table 1:**
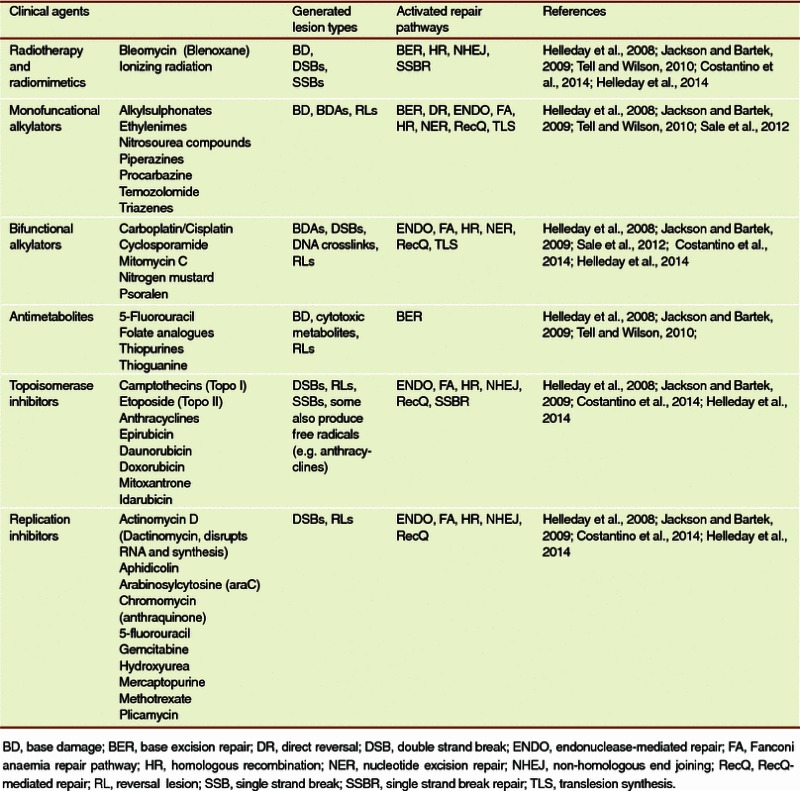
A partial list of DDR-inducing antineoplastic agents in medical oncology, the damage types and correspondingly activated repair pathways in human cells

Characterized with either “senescence-associated secretory phenotype” (SASP) (Coppe et al., 2008) or genome-wide “senescence-messaging secretome” (SMS) (Kuilman and Peeper 2009), damaged cells significantly alter surrounding microenvironments through robust secretion of soluble factors. As a full senescence response is not required to initiate this reaction program and acute stress-associated phenotype (ASAP) can appear prior to cellular senescence (Gilbert and Hemann, 2010), a more appropriate term for the chronic response that exerts long term effects after cell exposure to genotoxicity is a “DNA damage secretory program (DDSP)” (Sun et al., 2012). The DDSP is remarkable and distinct in several respects. First, the program is complex, with several hundred proteins induced to varying levels; Second, the program is robust, a large array of transcripts and their attendant proteins, are induced more than 10-fold; Third, there is both consistency and variability in the program depending on the cell type—e.g. fibroblast or epithelium; Fourth, multiple DDSP proteins are well-known to promote disease evolution particularly tumor progression, including proteases (MMPs), growth factors (AREG, EREG), pro-angiogenic factors (ANGPTL4, VEGF), and pro-inflammatory cytokines (IL-6, IL-8) (Coppe et al., 2008; Kuilman et al., 2008; Sun et al., 2012). The enhanced secretion of intracellularly synthesized factors to extracellular space promotes the proliferation and carcinogenesis of epithelial cells, increases angiogenesis, enhances epithelial to mesenchymal transition (EMT), accelerates the invasiveness of transformed cells, and stimulates the growth of xenografted tumors *in vivo* (Coppe et al., 2008; Sun et al., 2012). Particularly, DDSP occurs after treatment of cancer patients with DNA damaging chemotherapy and cause expression changes of oncogenic nodes in proximal surviving cells, thereby stimulating malignant phenotypes of the tumor (Coppe et al., 2010; Gilbert and Hemann, 2011; Sun and Nelson, 2012).

DDSP factors comprise several families of soluble and insoluble proteins. Such components can affect adjacent niches by activating various cell surface receptors and corresponding signal transduction pathways, actively causing multiple disorders. The secretion of IL-6, a pleiotropic pro-inflammatory cytokine increases markedly after DNA damage- and oncogene-induced senescence of mouse and human keratinocytes, melanocytes, monocytes, fibroblasts, and epithelial cells (Kuilman et al., 2008). Another upregulated interleukin by senescent cells is the IL-1 complex, whereby both IL-1α and -1β are significantly secreted by senescent endothelial cells, fibroblasts, and chemotherapy-induced senescent epithelial cells (Davalos et al., 2010). Chemokine IL-8 (CXCL-8), along with GROα and GROβ (CXCL-1, -2) are overexpressed upon senescence (Coppe et al., 2008). Intriguing production of both IL-6 and IL-8 depends on the expression and secretion of IL-1α, indicating an internal regulation of DDSP components (Davalos et al., 2010). As typical pro-inflammatory cytokines, IL-6 and IL-8 help reinforce senescence growth arrest in an autocrine manner (Acosta et al., 2008). Other growth factors and inflammatory cytokines including AREG, EREG, CXCL-3, CXCL-14, and IL-23A are also synthesized and secreted by DNA-damaged stromal cells (Sun et al., 2012).

Remarkably the matrix metalloproteinases (MMPs) family members are upregulated in senescent fibroblasts in particular stromelysin-1 and -2 (MMP-3, -10) and collagenase-1 (MMP-1) (Liu and Hornsby 2007). MMPs produced by senescent cells can regulate the activity of the soluble factors present in DDSP by cleaving MCP-1, -2, and -4 and IL-8 (Kessenbrock et al., 2010). Serine proteases secreted as part of DDSP are regulators of the plasminogen activation pathway: urokinase- or tissue-type plasminogen activators (uPA or tPA), the uPA receptor (uPAR), and inhibitors of these serine proteases (PAI-1 and -2) (Blasi and Carmeliet, 2002). Senescence-induced changes in cellular metabolism may alter TMEN by secreting certain ligand of Wnt/β-catenin pathway, namely WNT16B, as one of the critical DDSP factors that emerged recently. Once released from damaged stromal cells, WNT16B can remarkably promote cancer cell proliferation, migration, invasiveness, and more surprisingly, resistance to multiple cytotoxic treatments both *in vitro* and *in vivo* (Sun et al., 2012).

Interestingly, certain stromal components may have beneficial effects as a bright side such as tumor suppression, immune clearance, tissue repair (Ozdemir et al., 2014; Rhim et al., 2014), and particularly, chemosensitizing, for example, suppression of the secretion phenotype through NF-κB inhibition promoted resistance to chemotherapy in a mouse lymphoma model (Chien et al., 2011). Such a beneficial effect of senescent cells on pathology poses a paradox because wound healing and tissue repair decline with age. How to resolve such paradoxes? The transient presence of senescent cells may be beneficial, whereas their chronic contribution including creating local (and possibly systemic) inflammation, disrupting normal tissue structure and function, and fueling aging related pathologies in multiple recurrent malignancies, becomes undoubtedly deleterious in the late stage of lifetime (Campisi, 2013).

## Regulatory network of senescence and secretion phenotype

Mechanistically a DDR is indispensable for the increased secretion of a subset of DDSP factors, which initiates a signal amplification cascade to sense DNA damage, inducing cell cycle arrest and DNA lesion repair (Rodier et al., 2009). If the extent of DNA damage is irreparable, such a DDR becomes persistent so that distinct nuclear structures termed DNA-SCARS (DNA segments with chromatin alterations reinforcing senescence) form and the cell enters senescence but maintains a chronic, low level DDR (d’Adda di Fagagna, 2008; Rodier et al., 2011). Persistent DDR is necessary for a robust DDSP, while the upstream components of the DDR complex particularly ATM/NBS1/CHK2 transcriptionally regulate many DDSP elements through activating NF-κB and/or CCAAT-enhancer-binding protein-beta (C/EBP-β), nuclear factors persistently active in senescent cells (Kuilman et al., 2008; Acosta et al., 2008; Fumagalli and d’Adda di Fagagna, 2009). Deletion of C/EBP-β abrogates the enhancement of both IL-6 and IL-8, the most upregulated cytokines at senescence, and NF-κB knockdown significantly decreases the expression levels of majority (75%) DDSP factors (Acosta et al., 2008; Kuilman et al., 2008). In many cell types, an early response to senescence-inducing stimuli is increased expression of IL-1α, a plasma membrane-associated cytokine that binds its plasma membrane-associated receptor (IL-1R), which in turn initiates a signaling cascade that ultimately activates NF-κB (Orjalo et al., 2009).

As part of the acute secretory responses that occur in certain contexts, cytokines including IL-6 and Timp-1 are released in thymus after DNA damage, thereby creating a “chemoresistant milieu” that promotes the survival of a minimal residual tumor burden via protecting lymphoma cells from demise induced by doxorubicin (Gilbert and Hemann, 2010). Importantly, IL-6 production occurs as a result of p38 mitogen-activated protein kinase (p38MAPK) activation in tumor-associated endothelial cells rapidly after genotoxicity, an acute cytokine release that was also observed in treated human endothelial and hepatocellular carcinoma cells. Similarly, p38MAPK/MAPKAPK-2 pathway is activated in H_2_O_2_-treated human stem cells and is responsible for establishing an irreversible cell cycle arrest typical of senescence that is caused by permanent reactive oxygen species (ROS) production, where increased functional mitochondria are involved (Borodkina et al., 2014). Further, p38MAPK governs a posttranscriptional mechanism that sustains the protumorigenic DDSP, while inhibition of p38MAPK abrogates the tumor-promoting activities of cancer associated fibroblast (CAFs) and senescent fibroblasts (Alspach et al., 2014). Thus, p38MAPK is a TMEN-specific Achilles’ heel that may be exploited as a new therapeutic target. Although DNA damage signaling implicates the activity of p38MAPK (Köpper et al., 2013), a stress-responsive MAPK pathway component, it was reported that this kinase induces senescence by a DDR-independent mechanism (Freund et al., 2011). To date, even it is established that mitogenic signals ultimately activates the p53/p21 and/or p16INK4a/RB pathways (Campisi, 2013), how p38MAPK is engaged in response to genotoxic stress and how it relays signals to the downstream pathways to induce the expression of a full spectrum of DDSP effectors remains largely unknown, and continued inputs to completely clarify the entire and detailed reaction chain are needed.

Two microRNAs, namely miR-146a and miR-146b (miR-146a/b), negatively regulate the senescence-associated secretion of IL-6 and IL-8 (Bhaumik et al., 2009). These microRNAs are strongly upregulated in senescent human fibroblasts with a DDSP, dampening inflammatory cytokine secretion by reducing NF-κB activity through direct targeting IRAK1, eventually suppressing IL-6/8 secretion (Taganov et al., 2006). To the contrary, inhibition of IL-1R signals from downstream can curtail miR-146a/b level, demonstrating the implication of microRNAs in a negative NF-κB feedback loop of secretory regulation between IL-1α and miR-146a/b (Freund et al., 2010). The studies again highlight the essential role of NF-κB, and indicate miR-146a/b as central players of interleukin secretion within the DDSP network. More recently it was reported that stimuli inducing cell senescence, such as γ-irradiation and standard O_2_ concentration, can increase expression of miR-335, which correlates with senescence/aging in human mesenchymal stem cells (hMSCs) and inhibits their therapeutic actions through inhibition of AP-1 activity. *Vice versa*, forced expression of miR-335 resulted in early senescence-like alterations in hMSCs, including increased SA-β-gal activity and cell size, reduced cell proliferation capacity, augmented p16 expression, and more importantly, the development of a DDSP phenotype, indicating that miR-335 plays a key function in the regulation of reparative activities of hMSCs and that it not only mediates senescence/DDSP signaling but might be considered a marker for the therapeutic potency of these cells in clinical applications (Tomé et al., 2014).

**Figure 2:**
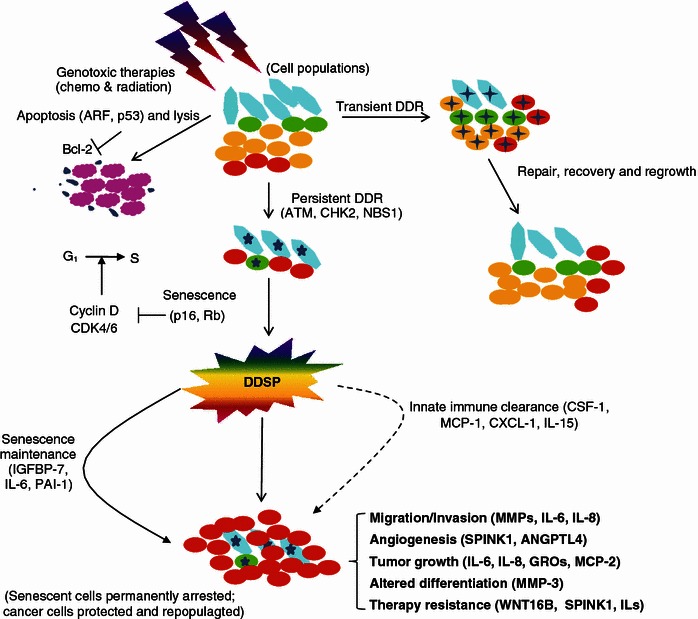
**Therapy-induced DDR activation triggers the DDSP program and exerts a profound impact to cancer phenotypes**. Upon therapeutic genotoxicity that mainly targets cancer cells, which are in active phases of turnaround and proliferation, cells within the TMEN have several responses. At minor damage, such as low doses of irradiation (0.5 Gy), DDR foci disappear within hours after complete DNA damage repair. In contrast, at higher doses (≥5 Gy), most damage foci in stromal cells persist for longer period, and cells enter senescence. Concurrently, majority of cancer cells are sensitized to such severe damage and directly go to apoptosis, while those with DDR-deficiency circumvent apoptosis and survive through treatment. High dosage of DNA damage usually triggers a persistent DDR engaging ATM, CHK2, and NBS1 which activates the cell cycle effectors p53/p16/RB, and leads to continuous and robust secretion of a large spectrum of proteins, dictated by a program coined as DDSP. A few secreted factors function in a cell-autonomous manner, such as IGFBP-7, IL-6, PAI-1, reinforcing senescence through a positive feedback loop that sustains the DDR. Several inflammatory cytokines (for example, CSF-1, MCP-1, CXCL-1, and IL-15), act in a cell non-autonomous way, and potentiate tumor regression by inducing the innate immune response that promotes tumor clearance. However, most components of the secretory phenotype promote cancer progression by enhancing survival, migration, invasiveness, and angiogenesis, accelerating cell repopulation, altering epithelial differentiation and causing epithelial-mesenchymal transition (EMT, e.g. IL-6, IL-8 and recently reported WNT16B and SPINK1). An advanced cancer phenotype, therapy resistance, hereby forms and once activated by such a program, cancer cells are more malignant and refractory to subsequent cycles of therapies. Colored cells: cyan, fibroblasts; green, smooth muscle cells; orange, benign epithelial cells; red, neoplastic epithelial cells; magenta, apoptotic cells; cross-shaped, cells undergoing transient DDR and in acute repair phase; star-shaped, cells that are permanently damaged, become senescent, and exhibit DDSP hallmarks

Thus, as evidenced by multiple studies, DNA damage is able to force surviving cells to enter senescence and engage a fairly complicated network that mediates the development of a signal transduction cascade. Damaged cells thereby exert comprehensive impacts to surrounding tissues with the accompanying, inherent, and conserved phenotype-DDSP, which can be turned on after clinical administration of genotoxic therapies against the extremely lethal human pathology, cancer (Fig. 2).

## Translational relevance and vistas

Cancer is a leading cause of global human mortality, and there is still much than less to do. Cells that are not intrinsically resistant to a drug will rewire their circuitry during treatment to become resistant, without any genetic or epigenetic changes at all (Bourzac, 2014). Various approaches including those to delay resistance acquisition through maintenance therapy are practiced, with a basic notion that resistance can be solved by increasing cancer cell exposure to therapy thereby killing them directly. Both pharmacokinetic and non-pharmacokinetic methods proved to be effective, however, neither side has taken into account of the far-reaching impact of therapeutically altered but biologically active TMEN, which once induced can significantly confound the disease control. Surviving cancer cells still proliferate during the intervals between treatments and this process of repopulation frequently results in treatment failure. Innovative strategies are eagerly expected to counteract with cancer cell repopulation during therapy, tackle tumor resistance, thus improving the overall treatment outcome.

### Cancer biology

Upon major DNA damage generated in cancer patients, apoptosis is the immediate response to chemotherapy, occurring within hours following therapy. As tumors that undergo apoptosis in response to therapy fail to display a significant residual tumor mass, it is conceivable that damage aftermath, mainly senescence, occurs at much slower kinetics, serving as a “standby” failsafe program, in case the first-line response is corrupted (Schmitt, 2003).

Emergence of prosurvival, progrowth, and proangiogenic factors derived from damaged TMEN is one of the conserved responses to genotoxic therapies, but in an off-target manner. Of note, multiple soluble factors are substantially released following DNA damage to cells *in* *vivo*, and paradoxically, such a secretory phenotype is formed more remarkably in proximal stromal cells than in the cancer population, which should be a major target of damage *per se* (Sun et al., 2012). For instance, drug treated endothelial cells release IL-6 and Timp-1, which promote the induction of Bcl-xl in adjacent lymphoma cells (Gilbert and Hemann, 2010). In such a case, proapoptotic signaling induced by the first salvos of chemotherapy on cancer cells is counteracted by anti-apoptotic signals emanating from the passively engaged vascular compartment in a damaged TMEN.

While the complete signal network starting from a DNA damage response to increased secretion still remains unclear, it involves the activation of stress responsive cascades—most notably the NF-κB signaling pathway. The NF-κB family of transcription factors plays indispensable roles in regulating expression of many SASP components. Constitutive blockade of NF-κB nuclear translocation through mutations in the IκBα gene, not only inhibited expression of multiple cytokines including the GRO family members, but also drastically attenuated WNT16B synthesis by the damaged prostate fibroblasts (Sun et al., 2012). On the other hand, intact NF-κB signaling is not only an essential pathway in many cancer entities, such as mucosa-associated gastric lymphoma, Hodgkin’s disease, and diffuse large-B-cell lymphoma, but can compromise the response to anticancer therapies (Schmitt 2003). Thus, the NF-κB complex seems to be implicated in both the development of a secretory phenotype of damaged stromal cells, and vital activities particularly survival of targeted cancer cells, each side is closely associated with acquired resistance. Similarly, the transcription factor C/EBP-β cooperates with IL-6 to activate the inflammatory network, while C/EBP-β depletion abrogates both IL-6 and IL-8 expression (Kuilman et al., 2008). Thus, novel agents with the ability to preferentially target NF-κB and C/EBP-β in human cells thereby dampening the development of a typical secretory phenotype will likely lead to clinical amelioration.

Poly (ADP-ribose) polymerase 1 (PARP-1) detects DNA lesions and promotes DNA repair, but can also mediate NF-κB activation upon genotoxic stress, as suppression of NF-κB activity and CCL2 secretion by the PARP-1 inhibitor 3-AB diminishes a secretome in senescent melanoma cells (Ohanna et al., 2011). Thus, the combination of temozolomide or fotemustine with PARP-1 inhibitors in clinical trials (Helleday et al., 2008) or with NF-κB inhibitors (e.g. sulfasalazine, in clinics for inflammatory bowel diseases) may confer potent anti-tumor efficacy and improve the therapeutic index by hypersensitizing melanoma cells to DNA damage while preventing the deleterious side effects. As a proof, PARP-1 inhibition in melanoma and cervical carcinoma lines enhanced *in vitro* sensitivity to temozolomide (Tentori et al., 2010) and promotes antitumor activity in ETS gene-rearranged PCa models (de Bono et al., 2011). These data provide strong support for the development of PARP1 or NF-κB blockage strategies in combination with the current anti-cancer drugs, and open new avenues for more effective therapeutic intervention.

Recently immunotherapeutics have entered clinics, largely on the basis of the recognition that several types of immune activities are correlated with tumor development. Although resistance-augmenting microenvironments represent a major barrier to effective elimination of multiple malignancies, combination regimens including synergistic chemoimmunotherapeutic approach that involves anti-CD20 (or anti-CD52) turns out to be a potent strategy for using conventional anticancer agents to alter the TMEN and promote the efficacy of targeted treatments (Coussens et al., 2013; Palucka and Banchereau 2014; Pallasch et al., 2014). Alternatively, some agonistic monoclonal antibodies, specific to certain molecules including those on the top list of DDSP factors or critical signaling nodes of damage response, have proved effectiveness through *in vitro* and *in vivo* studies (unpublished). Once approved to enter clinical trials, they will demonstrate the values in circumventing the side-effects of DNA-damaging treatments. In a long term, it benefits to develop medication that enhances DNA damage but sever the signaling pathway arising from DDR events to the downstream consequence, DDSP program development, thereby depriving cancer of acquired resistance and reducing disease incidence, progression and mortality.

### Clinical management

Cancer cell repopulation describes the continued proliferation of surviving cancer cells, most of which are usually cancer stem cells (CSCs) and have the capacity to regenerate the tumor, occurs during a course of fractionated radiotherapy or chemotherapy. As a result of influence from the TMEN that not only limits drug distribution but exerts multiple anti-treatment forces, repopulation often has a dominant and limiting effect on therapeutic index, and accelerated repopulation during successive therapy can lead to an initial response followed by tumor regrowth in the absence of intrinsic sensitivity change of cancer cells (Yu and Tannock, 2012; Patel et al., 2013; Yu et al., 2013).

**Figure 3:**
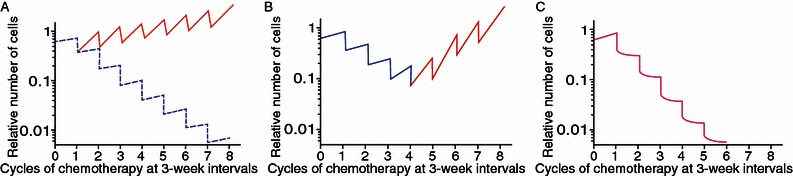
**Potential effects of repopulation on total cell number between therapeutic cycles and a proposed clinical solution to circumvent pathological consequences**. (A) Although a large portion of cancer cells is killed after each dose of administration, such as those given at 3-week intervals of radiation or chemotherapies, surviving cells can repopulate within a TMEN during the overall treatment period. Here, a constant rate of repopulation between doses is assumed, characterized by a doubling time of either 10 days (solid lines, red) or 2 months (dashed lines, blue). (B) Accelerating repopulation of surviving cancer cells frequently occurs between consecutive cycles, characterized by the indicated doubling times (blue line, cell number reduction phase; red line, increase phase). Such tendency can lead to cancer remission and regrowth, a phenomenon commonly observed in clinical practice, without any change in the cell intrinsic sensitivity to treatments. (C) Novel and innovative regimens integrating therapies that effectively target the DDSP phenotype of damaged TMEN (green arrows) have the advantage to curtail the potential of cancer cell repopulation. Ideally this should be applied consistently between successive courses to achieve optimal outcome in medical oncology

Upon chemotherapy tumor growth will follow a Gompertzian curve, and the rate of regrowth would be faster after shrinkage induced by treatment (Norton and Simon, 1986). In a model to mimic the responses of cancer cells during repopulation that occurs in courses of chemotherapy given at three-week intervals, it is reasonable to assume that the rate of repopulation remains constant but increases in the intervals between successive courses of treatment, whereas chemotherapy inhibits proliferation temporarily after treatment but triggers subsequent repopulation which accelerates between successive cycles of chemotherapy (Kim and Tannock, 2005). Increased cellular sensitivity to cytotoxic drugs generated by rapid proliferation might be countered by the tendency to select cells with acquired resistance conferred by enhanced secretion of DDSP factors from adjacent TMEN in the context of hypoxia. In clinical conditions, this problem may be technically solved by artful administration of anti-DDSP agents between cycles of therapy, a feasible and practical way to minimize the influence of cancer-promoting factors from DNA-damaged cells that develop a secretory phenotype (Fig. 3).

Repopulation depends on the activation of specific signaling pathways to promote cancer cell proliferation. In clinical trials, agents including small molecule inhibitors and antibody-based pharmaceuticals can be given concurrently or continually with conventional therapy (Hallek et al., 2014). One caveat is, the results of some trials may not be exciting, as the cytostatic effects of molecule-targeted agents might render tumor cells insensitive to cycle-active chemotherapy. Thus, it is worthwhile to conceive advanced clinical trials, in which adjuvant chemotherapy (cytotoxic) agents are administered but with enhanced specificity to proliferating cells, while novel drugs (cytostatic) targeting DDSP factors are used during therapy courses to shield influence from surrounding stroma. Thus, an ideal approach is to apply these types of treatments sequentially as a metronomic design (Sun and Nelson, 2012). The advantage of such a combinatory treatment is that before the next round of DNA-damaging therapy it will allow cancer cells to re-enter the cycle and regain sensitivity to cycle-active drugs, a regimen leading to improved therapeutic outcome.

On the other hand, despite tremendous resources being exploited in cancer biology and clinical oncology, there are progressively increasing failure rates, high cost, low bioavailability, poor safety, limited efficacy, and a lengthy design and testing process associated with cancer drug discovery and development. Exploring established non-cancer drugs for anticancer activity provides an opportunity to rapidly advance therapeutic strategies into clinical trials (Gupta et al., 2013). The impetus for development of cancer therapeutics that specifically manipulate the TMEN to deprive cancer cells of resistance conferred by surrounding niches, stems from the fact that many diseases share common molecular pathways and targets in the cell. For instance, multiple agents that inhibit PAPR1 activity have been approved by FDA and currently applied in non-cancer disease treatments where they demonstrated decent efficacy (Garber, 2013; Harrison, 2013); some antidiabetic chemicals like metformin can suppress the production of inflammatory cytokines generated by senescent cells and inhibit their secretory phenotype by interfering with IKK/NF-κB activation (Moiseeva et al., 2013). Clearly, such drugs have potential to be “repurposed” or “reprofiled” for cancer treatments. The efforts to employ advanced strategies to identify and implement current non-cancer drugs for cancer resistance control should be highly valued and encouraged, and we wish that drug repurposing will play a high-impact role in future anticancer therapies.

## Concluding remarks and future directions

Considerable progress is made in elucidating the underlying mechanisms of cancer resistance, with expected innovation in therapeutic design, both conceptually and practically. However, much is still in dark and many blanks remain unfilled. By nature, cancer is caused not just by bad cells or bad genes, but also by good ones not doing the right thing—an aspect of the disease that is highly complicated to study and to combat (Bourzac, 2014). The keys to success should include careful evaluation of the preclinical-clinical consequence of tissue damage generated by DNA-targeting therapies. Repopulation during fractionated anti-cancer therapies has long been recognized as an important cause of treatment failure. Recent findings outline a mechanism by which cytotoxic therapies given in cyclical doses can actually augment later treatment resistance and may open the door to new areas of research and to emerging therapeutic targets that implicate the DNA damage response program in certain cell types.

Preclinical trials are ongoing to evaluate strategies to manipulate the distinct phenotype of DNA-damaged cells, and a promising methodology is to combine the use of cytotoxic agents which shrink tumor mass by selectively inhibiting cell proliferation and that of novel drugs to diminish the development of DDSP of damaged TMEN during treatment intervals, a combined and integral approach. Although for new trials prudential scheduling of multiple agents between courses of therapy is not only necessary but essential, priority should be given to experimental and clinical studies of the process, which may be more complicated than thought. Last, but not least, translation of most scientific findings from laboratories to clinics has the potential and holds promise to improve the safety and efficacy of anticancer therapies, by successfully cutting the lifeline of cancer cell populations.

## Abbreviations

ASAP: acute stress associated phenotype
CAF: cancer associated fibroblast
C/EBP-β: CCAAT/enhancer binding protein
CSC: cancer stem cell
DDR: DNA damage response
DDSP: DNA damage secretory program
DNA-SCARS: DNA segments with chromatin alterations reinforcing senescence
hMSC: human mesenchymal stem cell
MDR: multiple drug resistance
MMP: matrix metalloproteinase
NF-kB: nuclear factor kappa-light-chain-enhancer of activated B cells
NK: natural killer
PARP-1: poly (ADP-ribose) polymerase 1
PCa: prostate cancer
p38MAPK: p38 mitogen-activated protein kinase
ROS: reactive oxygen species
SASP: senescence associated secretory phenotype
SMS: senescence messaging secretome
TMEN: tumor microenvironment
tPA: tissue type plasminogen activator
uPA: urokinase plasminogen activator
uPAR: uPA receptor
